# Phosphorylation of Ack1 by the Receptor Tyrosine Kinase Mer

**DOI:** 10.3390/kinasesphosphatases1030011

**Published:** 2023-07-10

**Authors:** Samantha Y. Hayashi, Barbara P. Craddock, W. Todd Miller

**Affiliations:** 1 Department of Physiology and Biophysics, Stony Brook University, Stony Brook, NY 11794, USA; 2 Department of Veterans Affairs Medical Center, Northport, NY 11768, USA

**Keywords:** Ack1, Mer, MHR, phosphorylation, autoinhibition, tyrosine kinase

## Abstract

Ack1 is a nonreceptor tyrosine kinase that is associated with cellular proliferation and survival. The receptor tyrosine kinase Mer, a member of the TAM family of receptors, has previously been reported to be an upstream activator of Ack1 kinase. The mechanism linking the two kinases, however, has not been investigated. We confirmed that Ack1 and Mer interact by co-immunoprecipitation experiments and found that Mer expression led to increased Ack1 activity. The effect on Ack1 was dependent on the kinase activity of Mer, whereas mutation of the Mer C-terminal tyrosines Y867 and Y924 did not significantly decrease the ability of Mer to activate Ack1. Ack1 possesses a Mig6 Homology Region (MHR) that contains adjacent regulatory tyrosines (Y859 and Y860). Using synthetic peptides, we showed that Mer preferentially binds and phosphorylates the MHR sequence containing phosphorylated pY860, as compared to the pY859 sequence. This suggested the possibility of sequential phosphorylation within the MHR of Ack1, as has been observed previously for other kinases. In cells co-expressing Mer and Ack1 MHR mutants, the Y859F mutant had higher activity than the Y860F mutant, consistent with this model. The interaction between Mer and Ack1 could play a role in immune cell signaling in normal physiology and could also contribute to the hyperactivation of Ack1 in prostate cancer and other tumors.

## 1. Introduction

Ack1 (Activated Cdc42-associated kinase 1) is a large (1038 residue), multidomain nonreceptor tyrosine kinase that was first identified by virtue of its interaction with the small G protein Cdc42 [1]. Ack1 is widely expressed in human tissues, with highest expression in the brain, spleen, thymus, and liver [2]. Ack1 has numerous cellular functions related to proliferation, cell survival, endocytosis, and protein trafficking [3,4]. Ack1 has been most heavily studied in the process of tumorigenesis. The gene encoding Ack1 is amplified or mutated in a number of primary human tumors, including lung, prostate, and ovarian tumors [3,5–7]. Ack1 overexpression in cancer cell lines increased cellular motility, invasiveness, and the ability to metastasize to the lung in a mouse model [5]. Genetic depletion or pharmacological inhibition of Ack1 point to a crucial role in cancer cell survival [8]. Taken together, the available data suggest that Ack1 and its upstream and downstream targets are potential targets for the design of anti-cancer drugs [9,10].

In normal cells, Ack1 can be activated by a variety of cell surface receptors, including G protein coupled receptors, integrins, and receptor tyrosine kinases (RTKs). Among the RTKs that have been implicated in Ack1 activation are EGFR, PDGFR, insulin receptor, IGF1R, Axl, LTK, ALK, and Mer [2,5,6,11,12]. The molecular details of how growth factor binding and RTK stimulation led to Ack1 activation have not been established in any of these cases. Ack1 possesses many potential sites for protein–protein interactions throughout its sequence [13]. From N-terminus to C-terminus, Ack1 has a sterile alpha motif (SAM) domain, a catalytic domain, an SH3 domain, a Cdc42/Rac-interactive (CRIB) domain, a clathrin-binding motif, a proline-rich region, a motif that shares homology with Mig6 (MHR), and a ubiquitin association (UBA) domain. The portion of Ack1 that binds to EGFR has been mapped to its C-terminal proline-rich and MHR regions [14]. Ack1 plays a significant role in the cellular turnover of EGFR [14–16]. For Axl, LTK, and ALK, Ack1 binding is promoted by the adaptor protein Grb2 [11], suggesting that direct RTK-Ack1 binding might not be obligatory for Ack1 activation.

Mer is a member of a family of RTKs designated the TAMs (named for the three members, Tyro3, Axl, and Mer). TAM receptor ligands include growth arrest specific-6 (Gas6) and protein S [17]. While increased TAM receptor expression is linked to cancer, loss of TAM signal transduction plays a role in autoimmune diseases [18,19]. TAM receptors are involved in the phagocytosis of apoptotic cells by macrophages; defective clearance of apoptotic cells is thought to contribute to the development of systemic lupus erythematosus (SLE) and other autoimmune disorders [18,19]. In mice, knockout of Mer blocks phagocytosis of apoptotic cells and leads to SLE-like phenotypes [20–22].

Mahajan et al. used mass spectrometry to identify Ack1 as a tyrosine-phosphorylated protein in Gas6-stimulated LNCaP prostate cancer cells [6]. Co-immunoprecipitation experiments showed that Ack1 interacts with the Mer receptor after Gas6 treatment of the cells. Expression of Mer increased Ack1 phosphorylation, but the molecular mechanism underlying this phenomenon remains unknown. Ack1 has also been reported to contribute to inflammation and autoimmunity [23–26], suggesting possible functional connections between Ack1 and Mer in normal physiology. Here, we investigated how Mer receptor expression leads to the phosphorylation and activation of Ack1. Our results point to a particularly important role for the C-terminal MHR in Ack1 in mediating this effect.

## 2. Results

**Figure 1.**
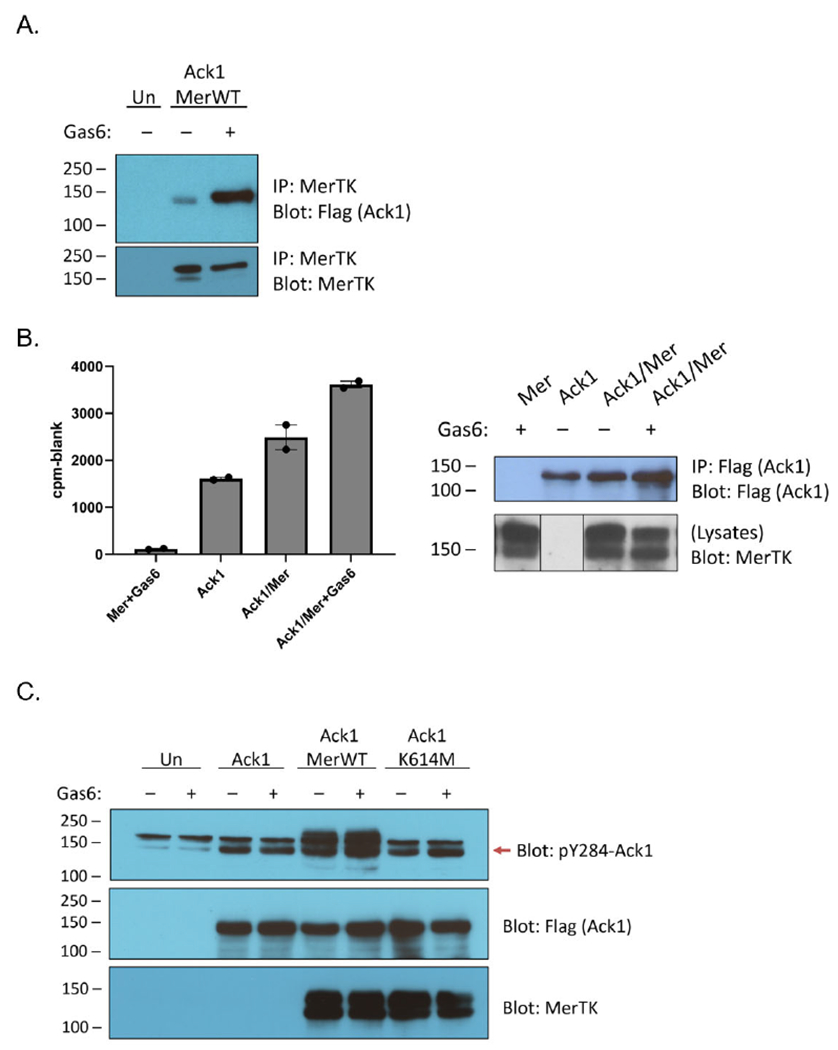
Interaction between Mer and Ack1. **(A)** HEK293T cells were transfected with Flag-tagged Ack1 and were either left unstimulated or treated with a final concentration of 120 ng/mL Gas6 for 15 min. Lysates of untransfected (Un) or Ack1-transfected cells (0.8 mg) were incubated with 1.4 μg anti-Mer antibody and 50 μL of a 50% slurry of Protein A-agarose at 4 °C overnight. The immunoprecipitated proteins were analyzed by SDS-PAGE with anti-Flag Western blotting. The membrane was then stripped and reprobed with anti-MerTK antibody. (**B**) HEK293T cells were transfected with Ack1 alone, Mer alone, or Ack1 plus Mer, and were either left unstimulated or treated with Gas6. (**Left**): Cell lysates (1 mg) were incubated overnight with immobilized Flag antibody. The activity of resin-bound Ack1 was determined in duplicate reactions using the phosphocellulose paper binding assay with WASP peptide and [^32^P]-ATP (SEM error bars). (**Right**): Western blot of the anti-Flag resin after incubation with Ack1 to check protein binding. The whole cell lysates were probed with anti-Mer to confirm expression. (Vertical lines indicate that these lanes were rearranged to match the order shown in the rest of the figure). (**C**) Lysates (100 μg) from unstimulated or Gas6-stimulated cells were analyzed by Western blotting with the indicated antibodies. The red arrow indicates the position of Ack1.

Ack1 and Mer have previously been observed to interact in the androgen-sensitive human prostate adenocarcinoma cell line LNCaP [6]. We confirmed the ability of the two proteins to interact by co-expressing Mer and Flag-tagged full-length Ack1 in HEK293T cells. Ack1 associated with immunoprecipitated Mer, and the interaction was stronger when cells were stimulated with Gas6, a ligand for Mer (Figure 1A). To determine whether the Mer–Ack1 interaction affected Ack1 kinase activity, we carried out anti-Flag immunoprecipitation reactions from HEK293T cells expressing Ack1 alone or Ack1 together with Mer. We performed in vitro kinase reactions on the immunoprecipitates by adding [γ-^32^P]-labeled ATP and a synthetic peptide substrate for Ack1 (derived from WASP, the Wiskott–Aldrich Syndrome Protein) [27]. Co-expression of Mer stimulated Ack1 activity by ~50%, whereas Mer plus Gas6 doubled Ack1 kinase activity (Figure 1B).

Co-expression of Ack1 with Mer has been shown to promote Ack1 phosphorylation [6]. To probe the mechanism of this process, we immunoprecipitated Ack1 from HEK293T cells co-expressing Mer and analyzed the immunocomplexes by Western blotting with anti-phospho-Ack1 (pY284) antibody. We previously used mass spectrometry to demonstrate that Y284 in the activation loop of Ack1 is the major site of enzyme autophosphorylation [28]. Co-expression with Mer increased levels of Y284 phosphorylation (Figure 1C). Co-expression with a kinase-inactive (K614M) mutant form of Mer did not increase Ack1 Y284 phosphorylation, indicating that the kinase activity of Mer was important for the effect (Figure 1C).

**Figure 2.**
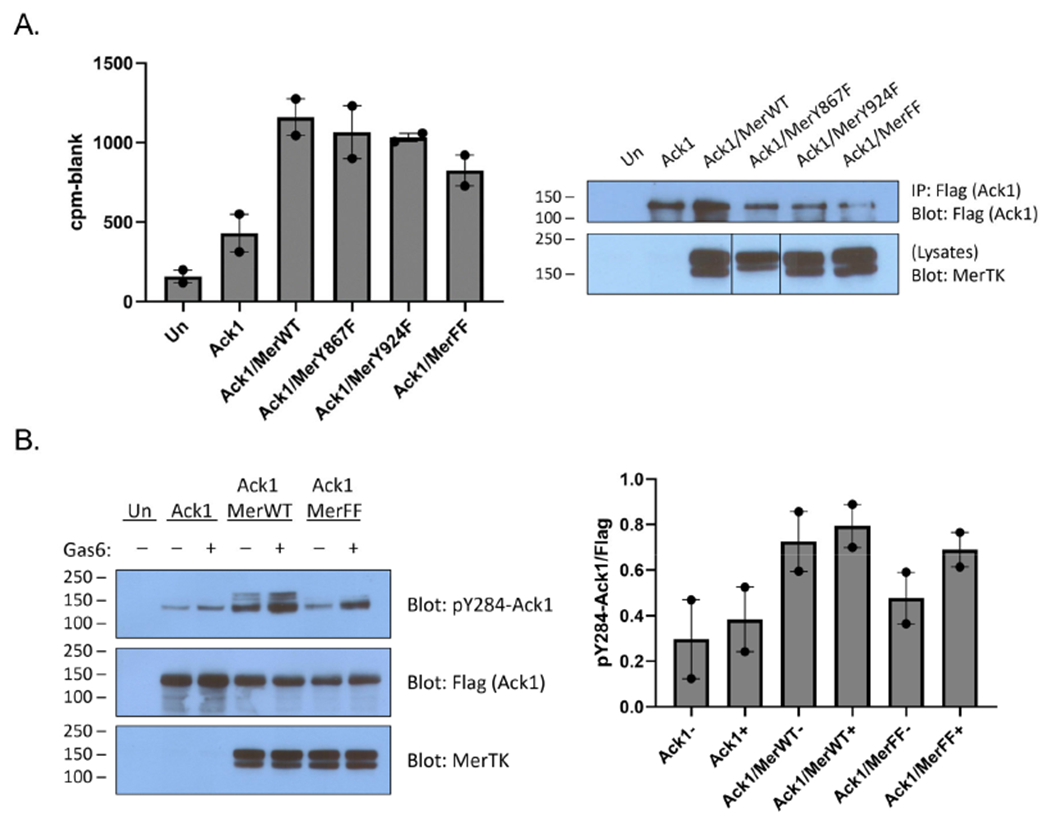
Mer C-terminal phosphorylation sites. (**A**) HEK293T cells were transfected with Ack1 alone or Ack1 together with Mer (wild-type or mutants; MerFF is the double Y867F/Y924F mutant). All cells were stimulated with a final concentration of 120 ng/mL Gas6 for 15 min. (**Left**): Lysates (1 mg) were incubated with anti-Flag resin overnight, and bound Ack1 was analyzed by in vitro kinase reactions, as described in the legend to Figure 1 (SEM error bars). (**Right**): Western blot of the anti-Flag resin after incubation with Ack1 to check protein binding. The whole cell lysates were probed with anti-Mer to confirm expression. (Vertical lines indicate that these lanes were rearranged to match the order shown in the rest of the figure). **(B)** Cells were transfected with Ack1 alone, Ack1 plus wild-type Mer, or Ack1 plus MerFF and were unstimulated or stimulated with 120 ng/mL Gas6 for 15 min. **(Left):** Lysates (100 μg) were analyzed by Western blotting with the indicated antibodies. **(Right):** Quantification of the Western blots, showing the ratio of signal for phospho-Ack1 (pY284) divided by the Flag signal for total Ack1 (*n* = 2, SEM error bars).

Mer has two primary sites of tyrosine phosphorylation, at Y867 and Y924 [29]. Although it is not known whether these sites play a role in Ack1 binding, there is indirect evidence from the Axl receptor kinase to suggest that Y867 could be involved. Axl and Mer are both members of the TAM family of receptor tyrosine kinases. Ack1 binding to Axl is mediated by Grb2, which binds to the proline-rich C-terminal region of Ack1 [11]. In a separate study, Grb2 was shown to bind to phosphorylated Y867 of Mer [29]. To test directly for a link between the C-terminal Mer phosphorylation sites and Ack1, we produced Y867F, Y924F, and Y867F/Y924F mutant forms of Mer. We co-expressed them with Flag-tagged Ack1 and analyzed Ack1 activity using the IP-kinase assay described above. The Tyr-to-Phe mutations caused modest decreases in Ack1 activity (relative to expression with wild-type Mer), but the differences were not statistically significant (Figure 2A). Co-expression with the Y867F/Y924F double mutant form of Mer reduced, but did not eliminate, Ack1 phosphorylation at Y284 (Figure 2B). These results suggested that interactions between the C-terminal phosphorylation sites of Mer and the Pro-rich region of Ack1 might not be the primary determinant of binding and led us to investigate alternative modes of interaction. In the sections below, we describe two lines of investigation: (1) testing for a direct phosphorylation of the Ack1 kinase domain by Mer kinase; and (2) testing for Mer-mediated phosphorylation of the C-terminal regulatory Mig6-homology region of Ack1.

**Figure 3.**
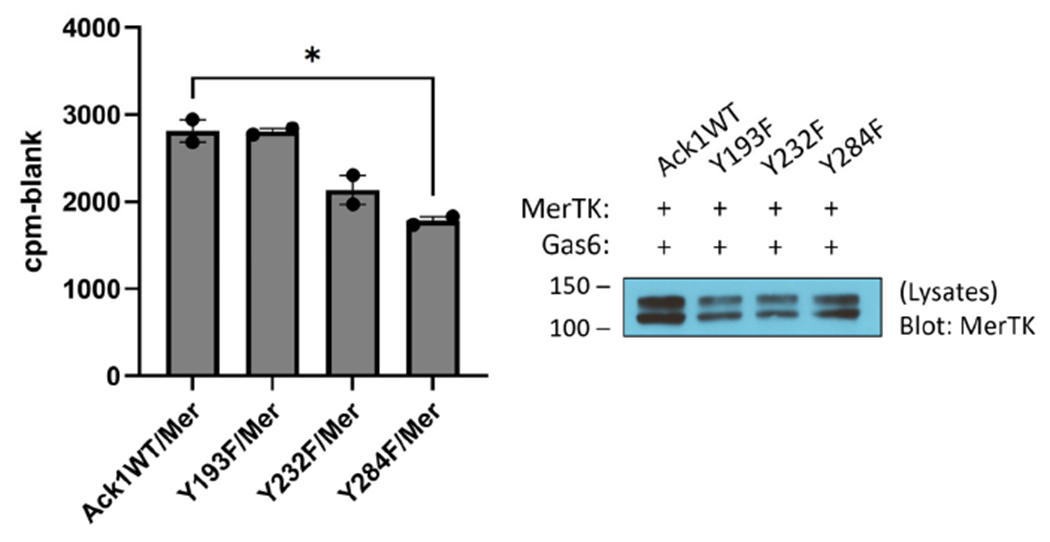
Mer phosphorylation sites in the Ack1 kinase domain. HEK293T cells were co-transfected with Mer plus wild-type or mutant forms of Ack1. All cells were stimulated with a final concentration of 120 ng/mL Gas6 for 15 min. **(Left):** Lysates (1 mg) were subjected to anti-Flag immunoprecipitation reactions overnight at 4 °C, and the activity of precipitated Ack1 was measured in an in vitro kinase reaction with WASP peptide and [^32^P]-ATP (SEM error bars) (* *p* ≤ 0.05, unpaired *t*-test). (**Right**): The whole cell lysates were probed with anti-Mer to confirm expression.

To test for the ability of Mer to phosphorylate Ack1 directly, we first expressed the His-tagged Mer kinase domain in *E. coli* cells together with YopH tyrosine phosphatase and GroEL [30]. We purified the Mer kinase by NiNTA chromatography, followed by a final anion exchange (MonoQ) column. We carried out Mer reactions with a purified Ack1 construct consisting of the kinase and SH3 domains. To identify sites of Mer-mediated tyrosine phosphorylation, we utilized mass spectrometry. We carried out parallel Ack1 phosphorylation reactions in the presence and absence of Mer and analyzed the results by LC/MS/MS (Figure S1). Two Ack1 sites were phosphorylated specifically in the reaction with Mer (i.e., not in the Ack1 autophosphorylation reaction): Y193 and Y232. These sites laid on the N- and C-terminal lobes of the Ack1 catalytic domain, respectively (Figure S2). To test the importance of these tyrosines, we produced Y193F and Y232F mutant forms of Flag-tagged full-length Ack1 and expressed them in HEK293T cells together with Mer. We carried out IP-kinase reactions on Ack1 isolated from these cells (Figure 3). The Y193F mutation had no effect on Ack1 activity, whereas the Y232F mutation led to a 20% reduction in Ack1 activity but was not statistically significant (Figure 3), suggesting that these sites were not likely to be the major contributors toward Mer-mediated Ack1 regulation. Mutation of Y284, the major autophosphorylation site in Ack1, led to a significant decrease in Ack1 activity (Figure 3).

**Figure 4.**
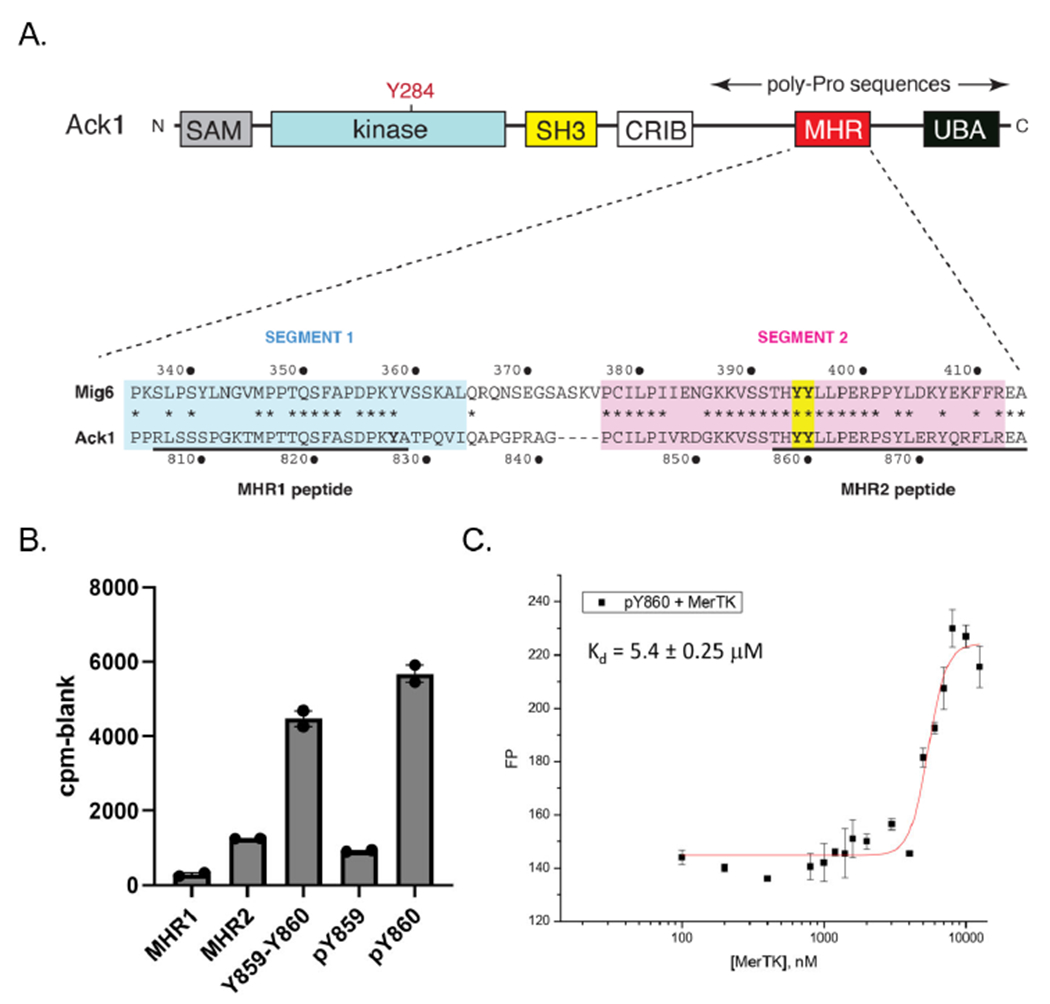
Ack1 MHR-derived peptides. (**A**) Position of the MHR domain in Ack1. The expanded sequences show an alignment between Mig6 and the MHR of Ack1, with Y859–Y860 in yellow. Asterisks indicate conserved residues. **(B)** Phosphorylation of MHR-derived peptides (100 μM) by Mer was measured using the phosphocellulose paper binding assay (SEM error bars). The sequences of the peptides are shown in Table 1. **(C)** Binding of fluorescein-labeled pY860 peptide to Mer kinase domain was measured by fluorescence polarization. The red line shows curve fitting using the logistic function in Origin (v.7).

Ack1 contains a C-terminal regulatory region with high homology to the EGFR inhibitor Mig6 (designated the Mig6 homology region, or MHR) [31] (Figure 4A). Phosphorylation of two adjacent tyrosines (Y859 and Y860) within this region by Src increases Ack1 activity, presumably by decreasing an intramolecular autoinhibitory interaction with the catalytic domain [31]. We tested a series of MHR-derived peptides as in vitro substrates of Mer (Figure 4B). Peptides derived from Segment 2 of the MHR, containing Y859 and Y860, served as efficient Mer substrates. We next tested two peptides containing tyrosine-phosphorylated Y859 or Y860 because the EGFR-Mig6 interaction has been shown to be dependent on a “priming” phosphorylation at the tyrosine corresponding to Y860 [32]. In these two peptides, the remaining tyrosine was unphosphorylated and, therefore, was available for Mer phosphorylation. Mer strongly preferred the peptide containing pY860 over the peptide containing pY859 (Figure 4B). We carried out steady-state kinetic measurements using a continuous spectrophotometric assay (Figure S3). The value of V_max_/K_m_ for peptide pY860 was ~3 times higher than the value for peptide pY859.

To confirm the interaction between pY860 and Mer, we attached fluorescein to the N-terminus of peptide pY860 and measured fluorescence polarization in the presence of increasing concentrations of Mer catalytic domain (Figure 4C). The peptide bound Mer with an apparent equilibrium dissociation constant (K_d_) of 5.4 μM. Binding of peptide pY859 to Mer was below the detection limit in this assay. These results suggested that Mer phosphorylation of Ack1 could follow a defined order, with Y860 preceding Y859.

**Figure 5.**
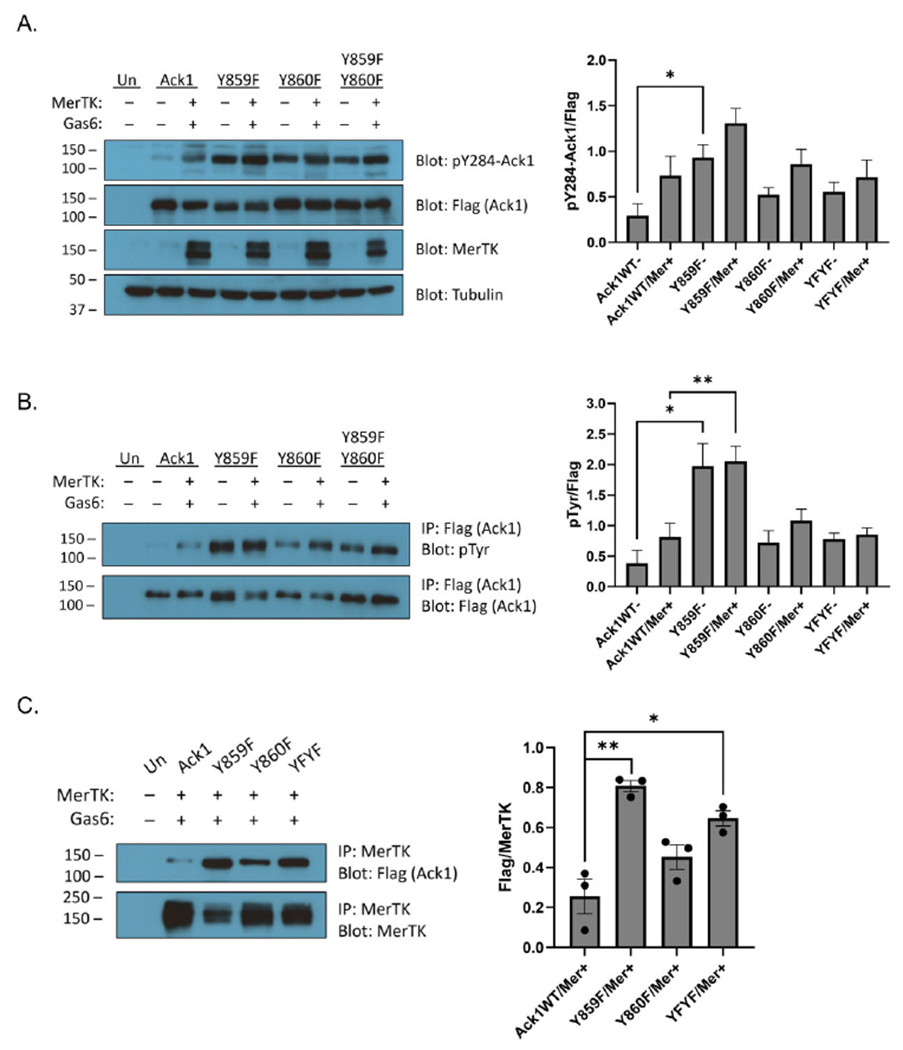
Mer phosphorylation of the Ack1 MHR. **(A)** HEK293T cells were transfected with Ack1 (wild-type or MHR mutants) in the absence or presence of Mer. Cells transfected with Mer were also stimulated with a final concentration of 120 ng/mL Gas6 for 15 min. (**Left**): Lysates (50 μg) were analyzed by Western blotting with the indicated antibodies. (**Right**): Quantification of the Western blots, showing the ratio of signal for phospho-Ack1 (pY284) divided by the Flag signal for total Ack1 (*n* = 4, SEM error bars) (* *p* ≤ 0.05, unpaired *t*-test). (**B**) Lysates (200 μg) were subjected to anti-Flag immunoprecipitation reactions for 3 h at 4 °C. (**Left**): Bound proteins were analyzed by anti-pTyr Western blotting. The membrane was stripped and reprobed with anti-Flag antibody. (**Right**): Quantification of the Western blots showing the ratio of signal for pTyr divided by the Flag signal for total Ack1 (*n* = 4, SEM error bars) (* *p* ≤ 0.05, ** *p* ≤ 0.01, unpaired *t*-test). (**C**) HEK293T cells were co-transfected with Mer plus full length Ack1 (wild-type or MHR mutants). All cells containing Mer were also stimulated with 120 ng/mL Gas6 for 15 min. (**Left**): Lysates (0.8 mg) were incubated with 1.4 μg anti-Mer antibody and 50 μL of a 50% slurry of Protein A-agarose at 4 °C overnight. The immunoprecipitated proteins were analyzed by SDS-PAGE with anti-Flag Western blotting. The membrane was stripped and reprobed with anti-Mer antibody. (**Right**): Quantification of the Western blots, showing the ratio of the Flag signal for total Ack1 divided by the Mer signal (*n* = 3, SEM error bars) (* *p* ≤ 0.05, ** *p* ≤ 0.01, unpaired *t*-test).

Next, we introduced Y859F, Y860F, and Y859F/Y860F mutations into full-length Ack1. We expressed the wild-type or mutant forms of Ack1 alone, or together with Mer. All cells containing Mer were also stimulated with Gas6. The Y859F, Y860F, and double Y859F/Y860F mutants showed increased Ack1 Y284 phosphorylation compared to the wild type, as measured by Western blotting (Figure 5A). These results were consistent with the known autoinhibitory roles of these tyrosines [31]. To confirm these findings, we immunoprecipitated the Ack1 constructs and carried out Western blotting with anti-pTyr antibody (Figure 5B). The mutants showed increased Ack1 tyrosine phosphorylation compared to wild-type Ack1, which corresponded with the Ack1 Y284 phosphorylation data (Figure 5B). The Y860F mutation, either alone or in combination with the Y859F mutation, blunted the increase in Ack1 tyrosine phosphorylation seen in Y859F (Figure 5B). Next, we immunoprecipitated Mer to analyze Flag/Mer binding (Figure 5C). The Y859F mutation led to an increase in co-immunoprecipitation with Mer relative to wild-type Ack1 (Figure 5C). The Y860F and Y859F/Y860F mutant forms of Ack1 bound less efficiently to Mer than the Y859F mutant (Figure 5C). These results were consistent with the peptide phosphorylation experiments (Figure 4), which suggested that tyrosine Y860 is an important initial site for phosphorylation.

## 3. Discussion

The Mer RTK plays an important role in normal physiology as the cell surface receptor for externalized phosphatidylserine (PtdSer), a trigger for recognition and phagocytosis of apoptotic cells [33]. Mer is found to be aberrantly expressed in non-small cell lung cancer and other malignancies, and the presence of Mer has been implicated in resistance to therapy with tyrosine kinase inhibitors [34]. The coupling between Mer and Ack1 kinases may be relevant to both of these situations. Ack1 was identified as a major tyrosine-phosphorylated protein in LNCaP prostate cancer cells that were stimulated with Gas6 to activate Mer [6]. The two proteins were shown to interact via coimmunoprecipitation in LNCaP cells, a result that we have recapitulated in HEK293T cells here (Figure 1A). Expression of a constitutively active mutant of Ack1 in LNCaP cells promoted anchorage-independent growth, and introduction of these cells into nude mice strongly accelerated the growth of tumors [6]. One important target for activated Ack1 in prostate cancer cells is the androgen receptor (AR). Phosphorylation of AR within its transactivation domain by Ack1 promotes binding of AR to specific androgen response elements of target genes, leading to androgen-independent gene expression and growth of prostate xenograft tumors [35]. It is important to note that Ack1 is activated by a number of other RTKs, in addition to Mer, including EGFR, PDGFR, AXL, ALK, and LTK [2,11,14,36]. Thus, the mechanistic details of the Mer–Ack1 connection can serve as a paradigm for understanding Ack1 activation by RTKs more broadly.

It is increasingly clear that Mer plays a crucial role in the macrophage-mediated phagocytosis of apoptotic cells [18,19,33]. There are multiple families of proteins that recognize PtdSer when displayed on the surface of apoptotic cells (a potent “eat-me” signal). The best characterized are proteins possessing a Gla domain (containing γ-carboxyl-glutamate). Gas6 has a Gla domain at its N-terminus and binds to Mer and other TAMs to promote tyrosine kinase activity. TAM receptors on the surface of phagocytes are critical for the PtdSer-driven clearance of apoptotic cells; mice lacking TAM receptors show multiple phenotypes related to systemic autoimmunity due to the failure of these processes [20,22]. Mer itself appears to be the most important of the TAM receptors in apoptotic cell clearance because knockout of Mer alone abolished phagocytosis of apoptotic cells [21]. Ack1 has recently been shown to play a role in autoimmunity [23]. Ack1 promoted the activation of Toll-like receptors (TLRs) in macrophages, and the Ack1 inhibitor AIM-100 mitigated the lupus symptoms of mice treated with a TLR7 ligand [23]. These results suggest that the Mer–Ack1 signaling pathway could play a role in autoimmunity.

To shed light on the connection between Mer and Ack1, we first confirmed previous studies [6] that demonstrated a complex between the two kinases (Figure 1A). The co-immunoprecipitation between Mer and Ack1 did not necessarily indicate a direct interaction; an adaptor protein such as Grb2 [11] could bridge the two proteins. Binding to Gas6-activated Mer stimulated the tyrosine kinase activity of Ack1 (Figures 1 and 2). This activation of Ack1 depended on the kinase activity of Mer (Figure 1C). Our data did not show a clear dependence of this effect on either of the best-characterized C-terminal autophosphorylation sites (Y867 and Y924) since mutation of these sites individually or in combination led to only modest decreases in Ack1 stimulation (Figure 2).

In the next set of experiments, we considered the possibility that Ack1 could serve as a direct substrate for Mer. There is precedent for cross-activation of Ack1 by other tyrosine kinases that phosphorylate Y284 in the activation loop of Ack1. In addition to serving as the major Ack1 autophosphorylation site [28], Y284 is phosphorylated by Src when the two kinases are co-expressed [37]. The SH2 and SH3 domains of Src are responsible for binding to Ack1. Src is necessary for the EGFR-mediated activation of Ack1 and stimulation of Ack1 turnover [37]. In our experiments, we used highly purified constructs consisting of the Mer kinase domain and the Ack1 kinase-SH3 domains to test for direct phosphorylation. We used tandem mass spectrometry to identify two additional Mer tyrosine phosphorylation sites (Y193 and Y232) within the kinase and SH3 domains of Ack1. These sites have not been reported previously as phosphorylation or autophosphorylation sites. However, mutation of these sites to phenylalanine did not cause a significant reduction in Ack1 kinase activity when the enzymes were co-expressed with the Mer receptor (Figure 3).

A limitation of the in vitro studies with the purified Ack1 kinase-SH3 construct was that several key regulatory regions of Ack1 were not present, including the C-terminal MHR domain (Figure 4A). In particular, tyrosines 827, 859, and 860 in the MHR have been shown in phosphoproteomic studies to be phosphorylated in multiple contexts: in BCR-Abl transformed cells, in response to insulin activation, and in EGF-stimulated HeLa or HEK293T cells [38–40]. Src also phosphorylates these tyrosines in the Ack1 MHR, leading to Ack1 activation, potentially by releasing the MHR from an autoinhibitory interaction with the Ack1 kinase domain [31]. In our initial experiments, we tested synthetic peptides containing these tyrosine motifs. A peptide containing Y827 (MHR1) was not a good substrate for Mer (Figure 4B). In contrast, peptides derived from the sequence surrounding Y859–Y860 were phosphorylated with good kinetics (Figures 4B and S3). A peptide containing phosphotyrosine at Y860 was phosphorylated (at Y859) efficiently by Mer (Figure 4B) and bound to the Mer kinase domain with a K_d_ of 5.4 μM (Figure 4C), suggesting that Mer phosphorylation of the MHR could follow a defined order, with Y860 preceding Y859. Alternatively, interactions within the Mer active site could orient Y859 to be a better target for phosphorylation than Y860 when Ack1 was bound.

We tested this idea in intact cells by co-expressing full length Mer with full length wild-type or unphosphorylatable mutants of Ack1 (Figure 5). The Y859F, Y860F, and Y859F/Y860F mutations all displayed more Ack1 Y284 phosphorylation and tyrosine phosphorylation than the wild type (Figure 5A,B). The MHR region of Ack1 is autoinhibitory [31]; thus, our interpretation is that these mutations may weaken the interaction between the Ack1 kinase and MHR domains and increase overall enzyme activity. These results suggested that Mer phosphorylation of the Ack1 MHR could explain the previously observed activation in LNCaP prostate cancer cells [6].

Compared to the Y859F mutation alone, the Y860F mutation (alone or in combination with Y859) led to a decrease in Ack1 phosphorylation at Y284 in the activation loop (Figure 5a), Mer-mediated Ack1 activation (Figure 5B), and co-immunoprecipitation with Mer (Figure 5C). These differences were consistent with the hypothesis that the phosphorylation of Ack1 by Mer (and potentially other kinases) occurs in a sequence. Y859 may have decreased affinity for Mer without prior Y860 phosphorylation, as suggested by the poor binding between peptide pY859 and Mer (Figure 4C). Y860 could be phosphorylated first, facilitating the subsequent phosphorylation of Y859. The Y859F-only mutation (in which Y860 is available for phosphorylation) had the most activity, regardless of Mer co-transfection/Gas6 stimulation (Figure 5A,B). On the other hand, mutation of Y860 (alone or in combination with Y859) did not eliminate binding to Mer (Figure 5C). This indicated that phosphorylated Y859/Y860 cannot be the sole determinant of Mer–Ack1 binding. The predominant effect of the Tyr-to-Phe mutations appears to be the release of the autoinhibitory effect of the MHR region. This could conceivably result in conformational changes in Ack1 that expose other domains for binding (e.g., the SH3, CRIB, or Pro-rich regions).

There is increasing evidence to demonstrate a role for Ack1 kinase in immune cell signaling pathways. Gene expression studies in colon cancer patients have shown a correlation between Ack1 expression and the extent of immune cell infiltration [24]. Ack1 is required for apoptosis induced by TRAIL, an apoptosis-promoting factor [25], and dysregulation of Ack1 is linked to abnormal apoptotic activity [26]. Ack1 promotes the activation of the Toll-like receptor (TLR) family members TLR4, TLR7, and TLR9 in macrophages and dendritic cells [23]. A small molecule inhibitor of Ack1 (AIM-100) reduced the TLR-mediated activation of macrophages, diminishing the lupus-like phenotype in a mouse model [23]. Given the strong involvement of Mer in macrophage-mediated phagocytosis of apoptotic cells and in autoimmune disorders, the connections between these two tyrosine kinases could present new avenues to be exploited for therapeutic development.

## 4. Materials and Methods

### Materials.

Phenylmethylsulfonyl fluoride, sodium vanadate, bovine serum albumin, dithiothreitol (DTT), poly (Glu, Tyr), fluorescein isothiocyanate, holo-Transferrin, and buffer components were purchased from Sigma-Aldrich, St. Louis, MO, USA. Horseradish peroxidase linked anti-rabbit and anti-mouse secondary antibodies were from Cytiva, Marlborough, MA, USA. SuperSignal West Femto Chemiluminescent Substrate and Pierce ECL Western Blotting Substrate were purchased from Thermo Scientific, Waltham, MA, USA. Nickel-nitriloacetic acid (Ni-NTA) resin was from Qiagen, Germantown, MD, USA. The following antibodies and resin were obtained from MilliporeSigma, St. Louis, MO, USA: anti-phosphotyrosine clone 4G10 (05-321), anti-phospho-Ack1 (pY284) (09-142), anti-γ-Tubulin (T6557), horseradish peroxidase–linked anti-FLAG produced in mouse (F1804), and EZview red anti-FLAG M2 affinity resin. Anti-Mer antibody for Western blotting was from R&D Systems, Minneapolis, MN, USA (AF891). Anti-Mer antibody for immunoprecipitation was from abcam, Cambridge, UK (ab184086).

### DNA constructs.

Plasmid pIRES2-EGFP Mer encoding full-length Mer was a gift from Dr. Raymond Birge (Addgene plasmid # 14998). The bacterial expression plasmid MER_HUMAN_D0, encoding the His-tagged Mer kinase domain, was a gift from Drs. John Chodera, Nicholas Levinson, and Markus Seeliger (Addgene plasmid # 79705). The mammalian expression vector encoding Flag-tagged full length Ack1 has been described previously [41]. The baculovirus vector encoding the Ack1 kinase and SH3 domain (residues Q115-D453) was generated by PCR amplification from the Ack1 cDNA and ligation into the BamHI and NotI sites of pFastbac-Htb (Thermo Fisher, Waltham, MA, USA). Site-directed mutagenesis was performed using the QuikChange XL II kit (Agilent, Santa Clara, CA, USA), and all mutations were verified by DNA sequencing.

### Cell culture and transfection.

HEK293T cells (from American Type Culture Collection, Manassas, VA, USA) were maintained in Dulbecco’s modified Eagle’s medium (Corning, Corning, NY, USA) supplemented with 10% fetal bovine serum (VWR, Radnor, PA, USA) and 100 IU/mL penicillin, 100 IU/mL streptomycin, and 250 ng/mL amphotericin B. Cells were transfected 24 h after plating with 2.5 μL of Mirus Transit LT1 per μg of DNA. Cells were harvested and lysed 48 h after transfection in buffer containing 25 mM Tris (pH 7.5), 1 mM EDTA, 100 mM NaCl, 1% NP-40, 10 μg/mL leupeptin, 10 μg/mL aprotinin, 200 μM PMSF, and 0.2 mM Na_3_VO_4_. *Spodoptera frugiperda* (Sf9) insect cells were maintained in Sf-900 II SFM medium (Gibco, Waltham, MA, USA) supplemented with 2.5% fetal bovine serum, 100 IU penicillin 100 IU/mL streptomycin, and 250 ng/mL amphotericin B (Corning).

For stimulation experiments, HEK293T cells were starved overnight 24 h after transfection in 0.1% BSA, 50 μg/mL holo-Transferrin, 1 g/L glucose Dulbecco’s modified Eagle’s medium, and 100 IU/mL penicillin, 100 IU/mL streptomycin 250 ng/mL amphotericin B. Cells were starved 48 h after transfection in 1 g/L glucose Dulbecco’s modified Eagle’s medium for 4 h, then stimulated with a final concentration of 120 ng/mL Gas6 for 15 min.

### Protein expression and purification.

Purification of the Ack1 kinase-SH3 protein from baculovirus-infected Sf9 cells was essentially as described for the isolated kinase domain [41]. Briefly, recombinant baculovirus was used to infect 600 mL of Sf9 cells at 1.8 × 10^6^ cell/mL. The infected cells were harvested after 3 days and lysed in a French pressure cell in buffer containing 50 mM Tris-HCl (pH 8.5), 1% NP-40, 100 mM NaCl, 5 mM β-mercaptoethanol, 5 μg/mL leupeptin, 5 μg/mL aprotinin, and 1 mM PMSF. The cell lysates were cleared by centrifugation at 6000 rpm for 10 min, filtered with a 0.8 μm filter, and applied to a 4 mL Ni-NTA column (Qiagen). The column was washed with buffer containing 20 mM Tris-HCl buffer (pH 8.5), 20 mM imidazole, 0.5 M NaCl, 10% glycerol, and 5mM β-mercaptoethanol. The His-tagged Ack1 kinase was eluted with buffer containing 100 mM imidazole. Fractions containing the Ack1 kinase domain were pooled, concentrated, and stored at −80 °C.

His-tagged Mer kinase domain was expressed in *E. coli* YGT cells co-expressing YopH tyrosine phosphatase and GroEL [30]. A 1L culture of YGT/Mer cells was grown at 37 °C to OD_600_ = 0.6. Expression was induced by addition of IPTG (0.5 mM), and the culture was grown overnight at 16 °C with shaking. The cells were harvested by centrifugation at 6000 rpm for 10 min. The cell pellets were washed with PBS then lysed in a French pressure cell in 35 mL of buffer containing 50 mM Tris-HCl (pH 8.5), 100 mM NaCl, 5 mM β-mercaptoethanol, 10 μg/mL leupeptin, 10 μg/mL aprotinin, 1 mM PMSF, and 1% NP-40. The cell lysates were clarified by centrifugation at 40,000× *g* for 30 min, filtered over a 5 μm syringe filter, and added to 3 mL Ni-NTA resin. After rocking 45 min at 4 °C, the mixture was centrifuged at 4200 rpm for 5 min. Batch washes were carried out with lysis buffer and Buffer A (20 mM Tris (pH 8.5), 500 mM NaCl, 10 mM imidazole, 10% glycerol, 5 mM β-mercaptoethanol). The resin was then loaded onto a 1.0 cm ID column and washed with 20 mL Buffer A and 50 mL Buffer B (20 mM Tris (pH 8.5), 10% glycerol, 5 mM β-mercaptoethanol). Mer was eluted with buffer B supplemented with 100 mM imidazole, and 1.5 mL fractions were collected and analyzed by SDS-PAGE. Peak fractions were dialyzed and further purified on a 1 mL MonoQ column (Cytiva) using a buffer of 20 mM Tris (pH8.0), 5% glycerol, 1 mM DTT, with a 20 mL gradient to 1 M NaCl. Peak fractions of Mer were stored at −80 °C.

### Immunoprecipitation and Western blotting.

For Western blotting, HEK293T cell lysates were resolved by SDS-PAGE, transferred to PVDF membranes, and probed with the appropriate antibodies. For anti-Flag immunoprecipitation (IP) experiments, cell lysates (0.2–1 mg total protein) were incubated with EZview red anti-FLAG M2 affinity resin for 3 h-overnight on a rotator at 4 °C. For MerTK IP experiments, cell lysates (0.8 mg) were incubated with 1.4 μg anti-Mer antibody (ab184086) and 50 μL of a 50% slurry of Protein A-agarose at 4 °C overnight. For all IP experiments, the resin was washed three times with lysis buffer, eluted with SDS-PAGE buffer, and analyzed by SDS-PAGE. The proteins were transferred to PVDF membrane for Western blot analysis.

For IP-kinase assays, cell lysates (1 mg protein) were incubated with 30 μL of EZview red anti-FLAG M2 affinity resin at 4 °C for 4 h-overnight, then washed three times with Tris-buffered saline. A portion of each sample was eluted with SDS-PAGE sample buffer and analyzed by anti-Flag Western blotting. The remaining sample was used for a radioactive kinase assay, carried out in duplicate. The immunoprecipitated proteins were incubated with 25 μL of reaction buffer (30 mM Tris (pH 7.5), 20 mM MgCl_2_, 1 mg/mL BSA, 400 μM ATP), 1 mM WASP peptide (sequence: KVIYDFIEKKKG) [27], and 50–100 cpm/pmol of [γ-^32^P] ATP at 30 ° C for 15 min. The reactions were quenched by addition of 45 μL of 10% trichloroacetic acid. The samples were centrifuged, and 35 μL of the supernatant was spotted onto Whatman P81 cellulose phosphate paper. After washing three times with 0.5% phosphoric acid, incorporation of radioactive phosphate into the peptide was measured by scintillation counting. The cpm for a blank reaction (no enzyme) was subtracted from the cpm for each sample to account for background activity.

### Peptide phosphorylation assays

Synthetic peptides and phosphopeptides were purchased from Genemed Synthesis Inc., San Antonio, TX, USA and were purified by semipreparative HPLC on a Vydac C18 column. Peptide phosphorylation was measured using the phosphocellulose paper binding assay with [γ-^32^P] ATP, as described above. For determination of Mer kinetic constants, a continuous spectrophotometric assay was used [42]. In this assay, the production of ADP was coupled to the oxidation of NADH, which was measured as a reduction in absorbance at 340 nm. Reactions were performed at 30 ° C in buffer containing 100 mM Tris (pH 7.5), 1 mM ATP, 1 mM phosphoenolpyruvate, 0.28 mM NADH, 89 units/mL pyruvate kinase, and 124 units/mL lactate dehydrogenase, with 1 μM purified Mer. Absorbance data were recorded every 8 s. Kinetic constants were determined by nonlinear fitting to the initial velocity vs. (substrate) curves using GraphPad Prism, San Diego, CA, USA (v.9).

### Fluorescence polarization.

Phosphotyrosine-containing peptides were fluorescently labeled by reaction with fluorescein isothiocyanate (5 molar equivalents) in 100 mM potassium phosphate buffer (pH 7.0) for 1 h at room temperature. The fluorescent peptides were purified by gel filtration chromatography on G15 Sephadex. Fluorescence polarization was measured in a Wallac1420 Victor2 instrument using 96-well half area black plates (Thermo Fisher, Waltham, MA, USA). The excitation filter was 485 nm, and the emission filter was 535 nm. Measurements were performed in volumes of 50 μL using a buffer of 50 mM Tris (pH 7.4), with peptide concentrations of 0.25 μM or 0.5 μM. The data were analyzed with Origin software (Origin v.7).

### Statistical analysis.

Unpaired, two-tailed, parametric *t*-tests were performed for all relevant data using GraphPad Prism (v.9).

## Supplementary Material

Supplementary figures

## Figures and Tables

**Table 1.** Sequences of Ack1 MHR-derived peptides. The tyrosines available to be phosphorylated are indicated in red.

Peptide	Sequence
MHR1	RLSSSPGKTMPTTQSFASDPKYA
MHR2	THYYLLPERPSYLERYQRFLREA
Y859–Y860	RDGKKVSSTHYYLLPE
pY859	RDGKKVSSTHpYYLLPE
pY860	RDGKKVSSTHYpYLLPE

## Data Availability

Not applicable.
