# Supplementary material for: Phosphorylation of Ack1 by the Receptor Tyrosine Kinase Mer
Source: Kinases Phosphatases. Author manuscript; Available in PMC 2023 Sep 2. (PMC10473914; doi:10.3390/kinasesphosphatases1030011)
Supplement: Supplementary figures [file NIHMS1926530-supplement-Supplementary_figures.docx]

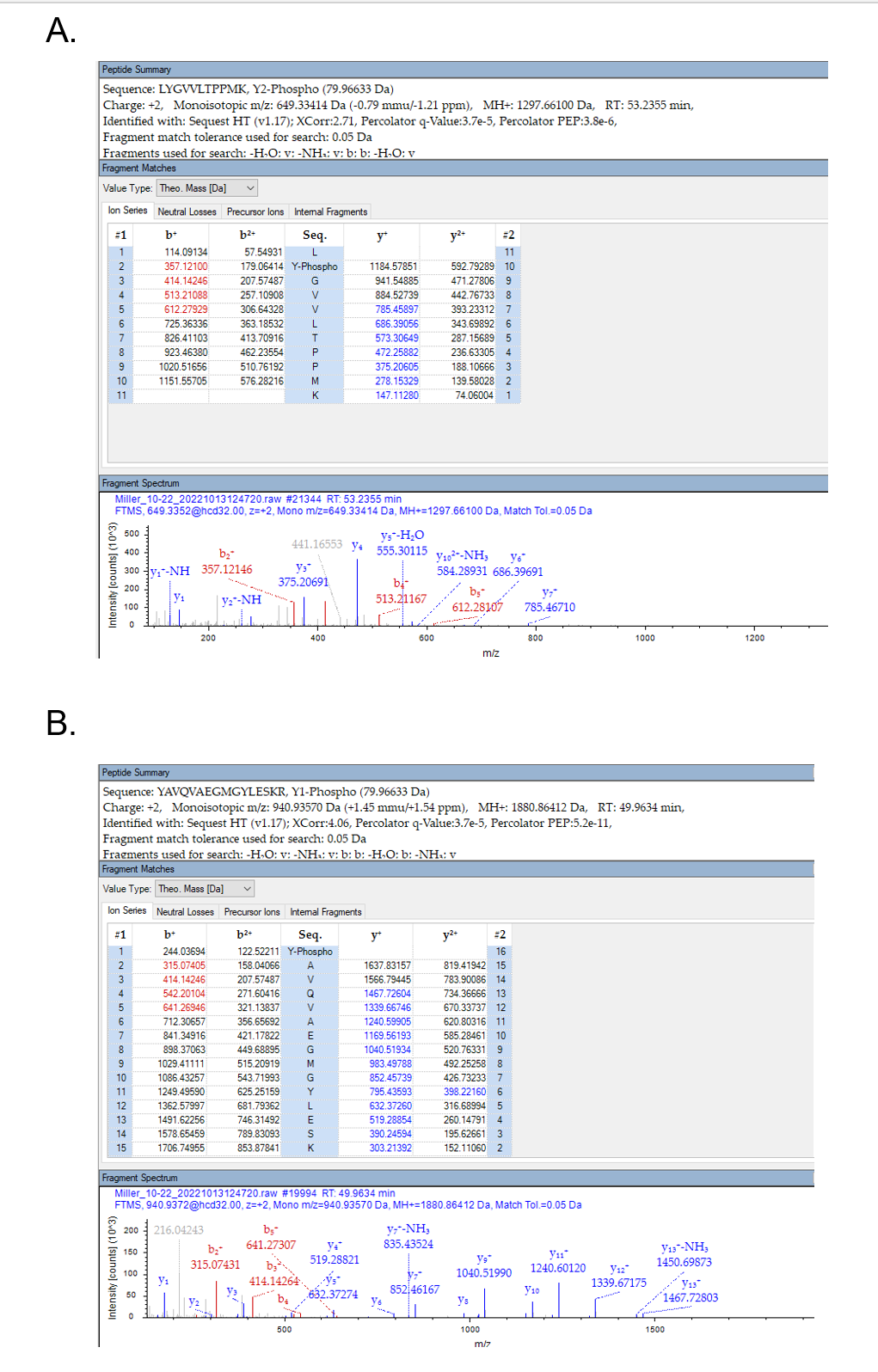


**Figure S1. Mass spectrometry analysis of Ack1 peptides phosphorylated by Mer.** These peptides were tyrosine phosphorylated in the reaction with Mer, but not in the Ack1 autophosphorylation reaction. (A) LC/MS/MS analysis of peptide containing Y193. Fragment matches are at the top, and a sample fragmentation spectrum is shown below. (B) LC/MS/MS analysis of peptide containing Y232.


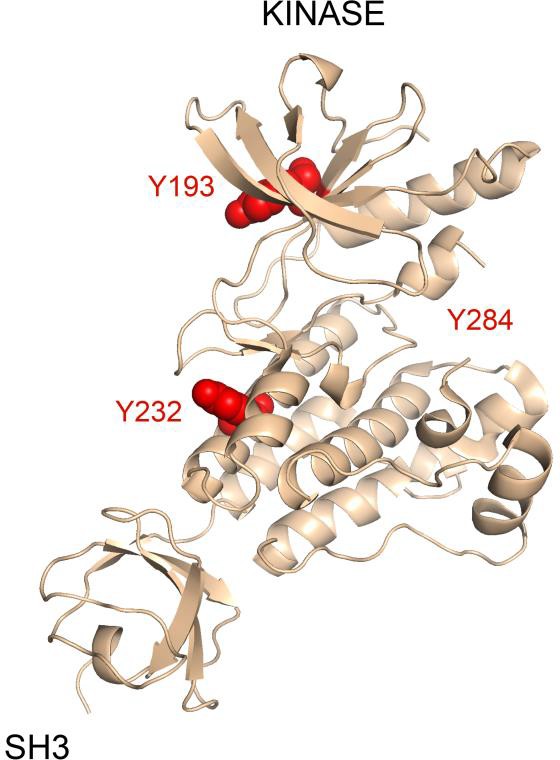


**Figure S2. Structure of Ack1 kinase and SH3 domains.** Y193 and Y232 are shown as red spheres on the structure of Ack1 (pdb code = 4HZS). Y284 in the activation loop is not resolved in this structure.


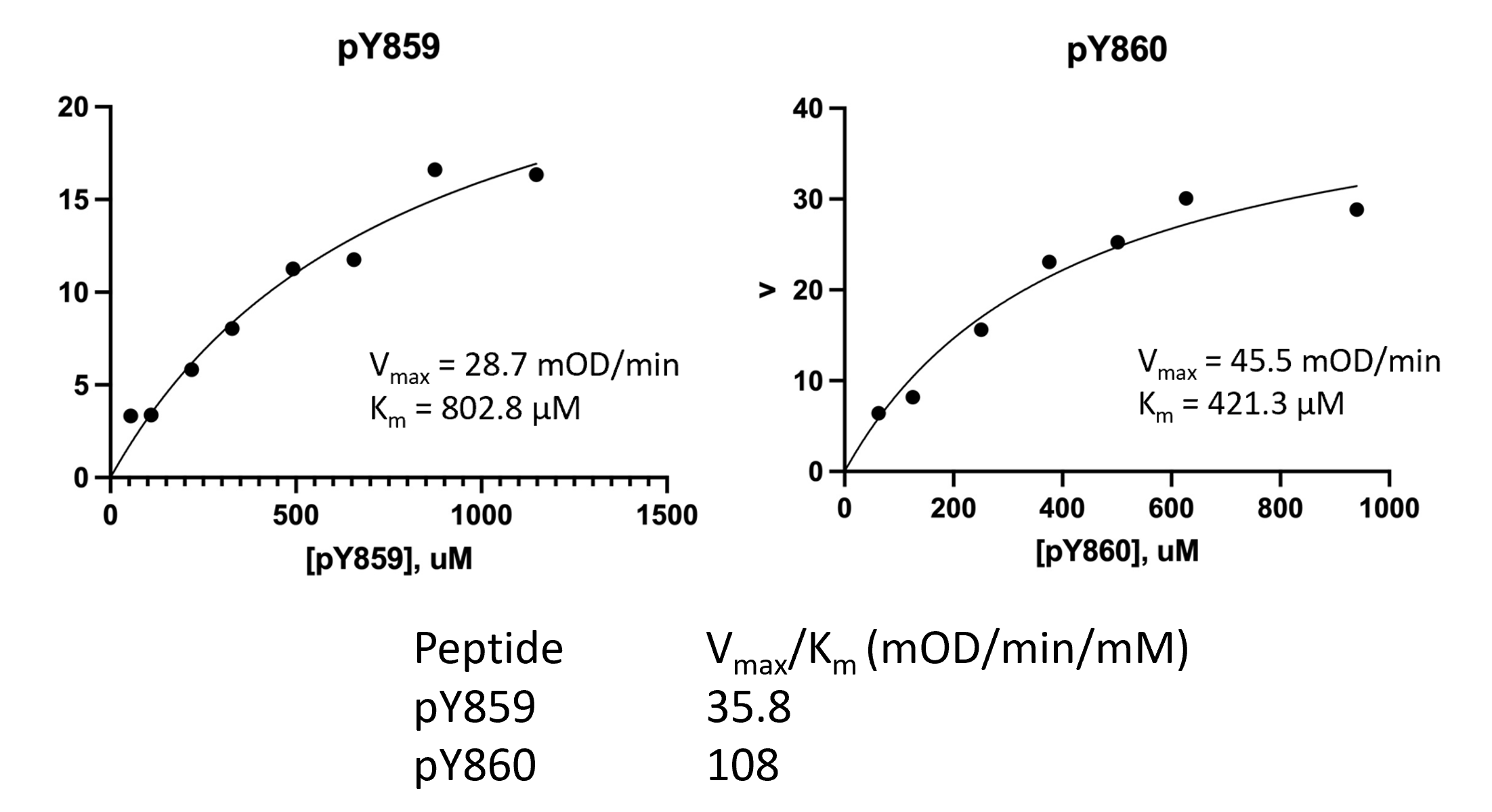


**Figure S3. Mer phosphorylation of Ack1 MHR phosphopeptides.** Steady state kinetic measurements were performed with the continuous spectrophotometric assay. Curve fitting was performed with GraphPad Prism (v.9), and the kinetic constants are shown.
